# Computational Prediction and Analysis of Breast Cancer Targets for 6-Methyl-1, 3, 8-Trichlorodibenzofuran

**DOI:** 10.1371/journal.pone.0109185

**Published:** 2014-11-03

**Authors:** Kumaraswamy Naidu Chitrala, Suneetha Yeguvapalli

**Affiliations:** Department of Zoology, Sri Venkateswara University, Tirupati, India; Wake Forest University, United States of America

## Abstract

Breast cancer is one of the most known cancer types caused to the women around the world. Dioxins on the other hand are a wide range of chemical compounds known to cause the effects on human health. Among them, 6-Methyl-1,3,8-trichlorodibenzofuran (MCDF) is a relatively non toxic prototypical alkyl polychlorinated dibenzofuran known to act as a highly effective agent for inhibiting hormone-responsive breast cancer growth in animal models. In this study, we have developed a multi-level computational approach to identify possible new breast cancer targets for MCDF. We used PharmMapper Server to predict breast cancer target proteins for MCDF. Search results showed crystal Structure of the Antagonist Form of Glucocorticoid Receptor with highest fit score and AutoLigand analysis showed two potential binding sites, site-A and site-B for MCDF. A molecular docking was performed on these two sites and based on binding energy site-B was selected. MD simulation studies on Glucocorticoid receptor-MCDF complex revealed that MCDF conformation was stable at site-B and the intermolecular interactions were maintained during the course of simulation. In conclusion, our approach couples reverse pharmacophore analysis, molecular docking and molecular dynamics simulations to identify possible new breast cancer targets for MCDF.

## Introduction

Dioxins are the persistent organic pollutants that are ubiquitous in soils, sediments, air, and animal tissues. Polychlorinated dibenzo-*p*-dioxins and furans are the widely distributed pollutants known to induce several toxic responses such as immunosuppression, carcinogenicity and disruption of reproductive, nervous and endocrine systems. Around 90% or more exposure to these contaminants by humans is through food [1]. Among the dioxins, 2,3,7,8-Tetrachlorodibenzo-p-dioxin (TCDD) a polychlorinated aromatic hydrocarbon is an ubiquitous environmental contaminant known to affect human health. It is the most toxic compound released as a product of waste incineration, herbicide overuse, paper chlorination and polyvinylchloride plastic production [2]. 6-Methyl-1,3,8-trichlorodibenzofuran (MCDF) a prototypical alkyl polychlorinated dibenzofuran on the other hand is a related compound to TCDD which is relatively nontoxic [3].

MCDF is known to interact with aryl hydrocarbon receptor (AhR), a transcription factor belonging to the basic helix-loop-helix/Per-ARNT-Sim (bHLH-PAS) family [4]. Study on AhR showed that it can be a potential drug target for estrogen receptor (ER)-positive breast cancer and relatively non-toxic 6-methyl-1,3,8-trichlorodibenzofuran (MCDF) is a highly valuable agent to inhibit hormone responsive breast cancer growth in animal models [5]. Previous studies showed that MCDF is used as an agent in the treatment of breast cancer by inhibiting 17β-estradiol (E2)-induced cell proliferation, tumor growth and prevents E2-induced increase in ER and progesterone receptor [3] [6].

Being an agent in the treatment of breast cancer, prediction and interaction analysis of new protein targets (having a direct or indirect role in Breast Cancer) for MCDF can elucidate its future therapeutic efficiency against Breast Cancer. Predicting new molecular targets for an individual molecule using experimental approaches is a time-consuming and expensive process. An alternate approach for this is using computational techniques. Among the computational techniques, virtual screening is the best one in such scenario. Present study aims at prediction and analysis of proteins (having a role in Breast Cancer) with three dimensional structures in Protein databank having theoretical binding sites for MCDF.

## Materials and Methods

An approach has been used to find the new Breast Cancer protein target for MCDF. This includes optimization, reverse pharmacophore mapping, validation, molecular docking and molecular dynamics of docked target protein – ligand complex.

### Molecule preparation

**Figure 1:**
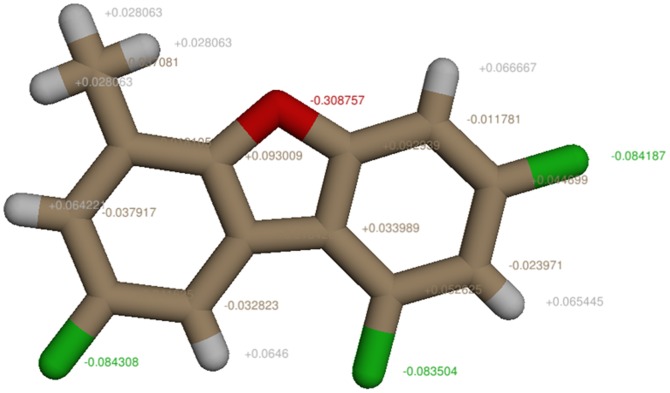
MCDF molecule with added Gasteiger partial charges using Chimera 1.6.2.

The structure of 6-methyl-1,3,8-trichlorodibenzofuran (MCDF) was downloaded from Pubchem compound database [7] (Compound ID: 114900). The downloaded MDCF structure was optimized by assigning Gasteiger partial charges with AMBER ff99SB force field and converted into mol2 format using Chimera 1.6.2 (Fig 1) [8].

### Pharmacophore mapping

PharmMapper is a web-based tool to predict potential drug targets against any given small molecule using ‘reverse’ pharmacophore mapping approach. It predicts the best mapping poses for a given query molecule against all the pharmacophore models in PharmTargetDB using ligand-protein reverse docking approach. Later, it lists the top *N* best-fitted hits with their respective aligned poses and target annotations [9]. The optimized structure of MCDF in mol2 format was submitted to PharmMapper (http://59.78.96.61/pharmmapper/; access date: September 12, 2012) for prediction of proteins with three dimensional structures in the Protein databank having binding sites to MCDF. The search started using the maximum generated conformations as 300 by selecting the ‘‘Human Protein Targets Only” option and default value of 300 for number of reserved matched targets.

### Molecular docking

Further, to characterize the novel binding sites in the predicted protein target we used AutoLigand from AutoDockTools. AutoLigand calculate the potential binding affinity of a protein based on total binding energy per volume for a fill, using the AutoDock force field [10]. The crystal structure of the antagonist form of Glucocorticoid Receptor (PDB code: 1NHZ) was downloaded from protein data bank (http://www.rcsb.org). By enclosing the entire structure in a 1.0 Å grid search space and generating many different-sized fill volumes using 10 to 210 fill points, novel binding sites were predicted. Next, using Autodock 4.2, we docked MCDF onto the crystal structure of Glucocorticoid Receptor. To generate the input files for docking, we employed the PyRx program [11]. The grid points were set as 126×126×126 with the spacing value at 0.375 on the novel binding site predicted by AutoLigand while the grid center was placed at 3.510, 20.165 and 0.433. The following settings were used for molecular docking using empirical free energy function and Lamarckian genetic algorithm (LGA): maximum number of energy evaluations: 2,500,000, initial population of randomly placed individuals: 150, maximum number of generations: 27,000, mutation rate: 0.02, crossover rate: 0.8 and elitism value (number of top individuals to survive to next generatione):1. For the local search, Solis and Wets algorithm was used with a maximum of 300 iterations per search. For all the other parameters none mentioned we used default values.

### Molecular dynamics simulation

The obtained complex of MCDF with crystal structure of the antagonist form of Glucocorticoid Receptor (MCDF-1NHZ complex) was subjected to Molecular dynamics (MD) simulations using GROMACS 4.5.5 with the standard GROMOS96 43A1 force field and the flexible SPC water model [12], [13]. The topology parameters for MCDF were generated using the PRODRG program [14]. The initial MCDF-1NHZ complex was immersed in a periodic water box of truncated dodecahedron shape (1 nm thickness) and neutralized with four Na+ counter ions. Particle mesh Ewald method was used to calculate the electrostatic energy [15]. Cut-off distances for the calculation of the Coulomb and vander Waals interaction were 1.0 and 1.0 nm, respectively. Energy minimization was done using a steepest decent algorithm with a maximum step size of 0.01 nm. The tolerance was 1000 kJ/mol/nm. The system was subjected to equilibration at 300 K temperature and 1 bar pressure. Finally, the full system was subjected to 30 ns MD and the atom coordinates were recorded every 2 ps during the simulation for later analyses.

### Molecular dynamics trajectories analysis

Comparative structural deviations in protein (1NHZ) and protein-ligand complex (MCDF-1NHZ complex) during the simulations were analyzed. g_rms, g_rmsf, do_dssp built-in functions of GROMACS package were used to compute the root mean-square deviation (RMSD), root mean-square fluctuation (RMSF), secondary structure calculation etc. Respective graphs obtained from molecular dynamics simulation were plotted using GRACE software (http://plasma-gate.weizmann.ac.il/Grace/).

## Results and Discussion

### Potential Protein Targets for MCDF

From the results of PharmMapper search, eight potential protein receptors for MCDF which are associated with Breast Cancer were identified. Respective role of the eight potential protein receptors in Breast Cancer was shown in the Table 1 given below. Their respective pharmacophore models were provided in the File S1. Among the eight potential protein receptors, Crystal Structure of the Antagonist Form of Glucocorticoid Receptor (PDB ID: 1NHZ) with highest PharmMapper Fit score (Table 1) was selected for further analysis.

**Table 1:** Respective breast cancer role for the potential targets of MCDF predicted using reverse pharmacophore approach.

PDB ID	Protein name	PharmMapper (Fit score)	Role in Breast Cancer	Reference
1NHZ	Glucocorticoid receptor	3.871	Accumulation in the cytoplasm of tumoral cells is associated with Breast cancer progression.	[17]
1S9J	Dual specificity mitogen-activated protein kinase kinase 1	3.786	Key molecule in regulating breast cancer growth and apoptosis.	[18]
1DB1	Vitamin D nuclear receptor	3.463	Induction of cell cycle arrest and apoptosis in breast cancer cells by 1,25(OH)_2_D_3_ and other VDR agonists is dependent on its expression.	[19]
1E3K	Progesterone receptor	3.35	Inhibits proliferation of human breast cancer cells.	[20]
2Q2Z	Kinesin-like protein KIF11	3.268	Elevated levels of mRNA expression seen in breast tumor samples	[21]
1DKF	Retinoid X receptor-Alpha	3.114	A potential therapeutic target in breast cancer.	[22]
1Q3D	Glycogen synthase kinase-3 Beta	3.027	Inactivation may leads to securin accumulation in breast cancers.	[23]
1P49	Steryl-sulfatase	3	Responsible for the maintenance of high estrogen levels in breast tumor cells	[24]

### Binding site prediction and docking calculations

**Figure 2:**
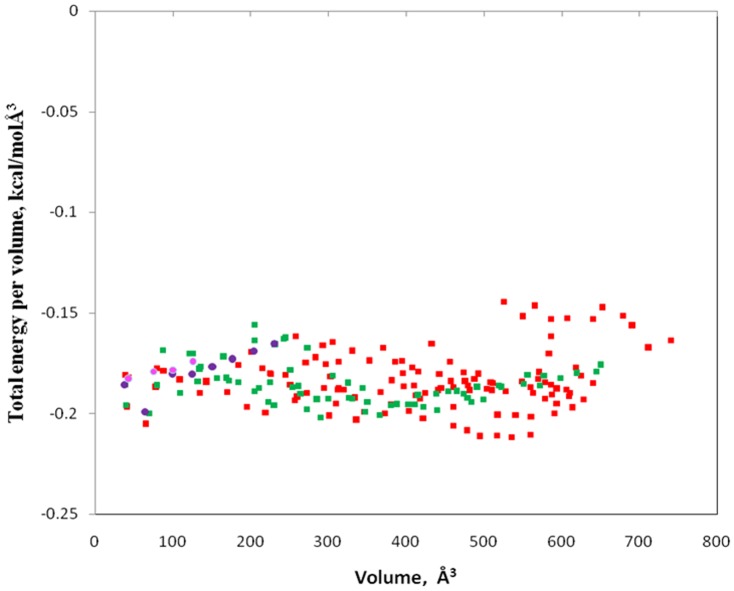
A plot of AutoLigand results of total energy per volume (kcal/molÅ^3^) versus volume (Å^3^), using fill sizes from 10 to 210 Å^3^. The results include two novel binding sites plotted with red and green squares and a few sets of points plotted in blue and pink circles representing small cavities.

**Figure 3:**
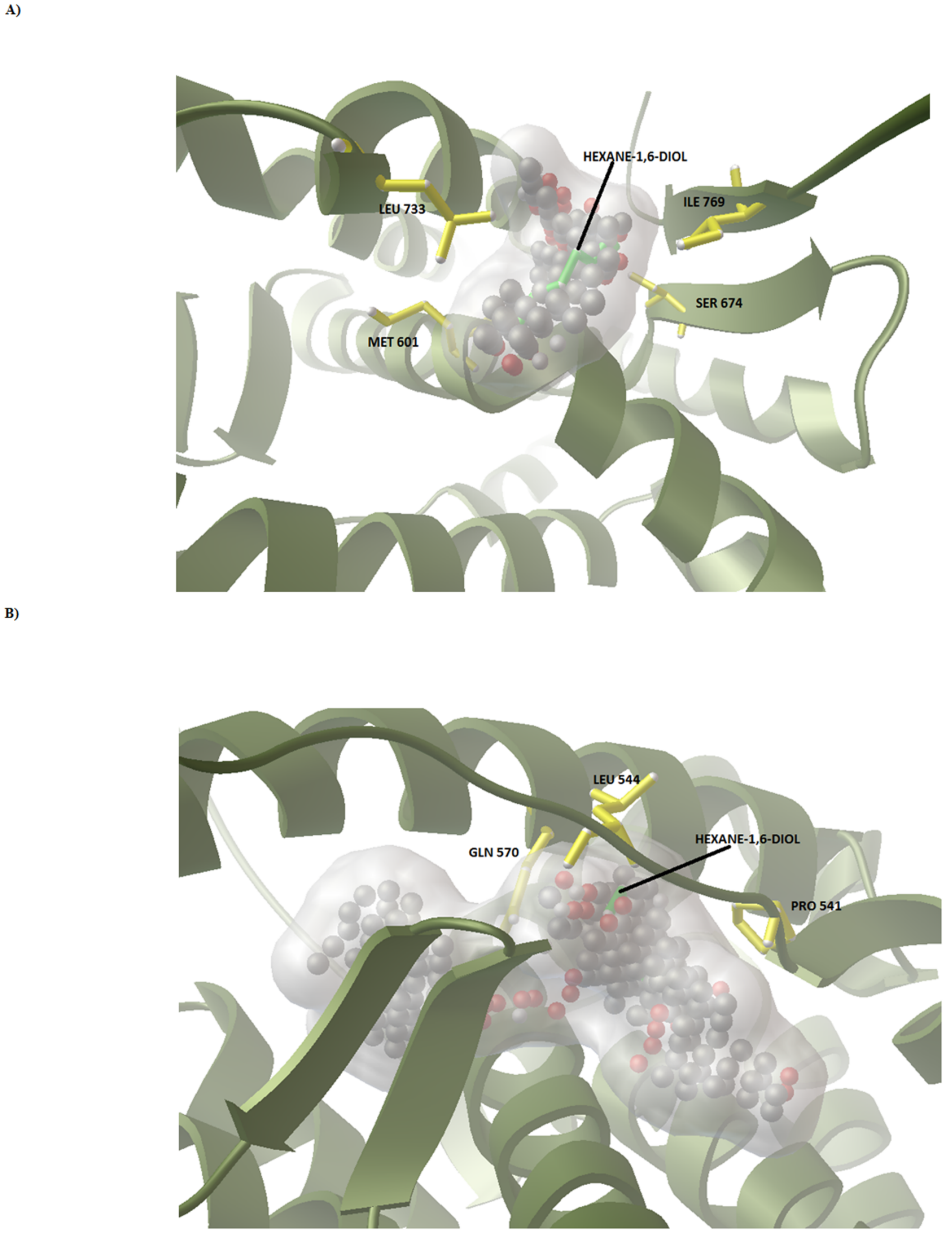
Potential small molecule binding site identified on the Glucocorticoid Receptor. (A) Represent the Auto Ligand fill at Site-A (B) Represent the AutoLigand fill at Site-B. Hexane-1,6-Diol was represented in green colored sticks, Glucocorticoid receptor was represented in green colored cartoons and its residues near the fill was represented in yellow colored sticks, fill was represented in balls.

**Figure 4:**
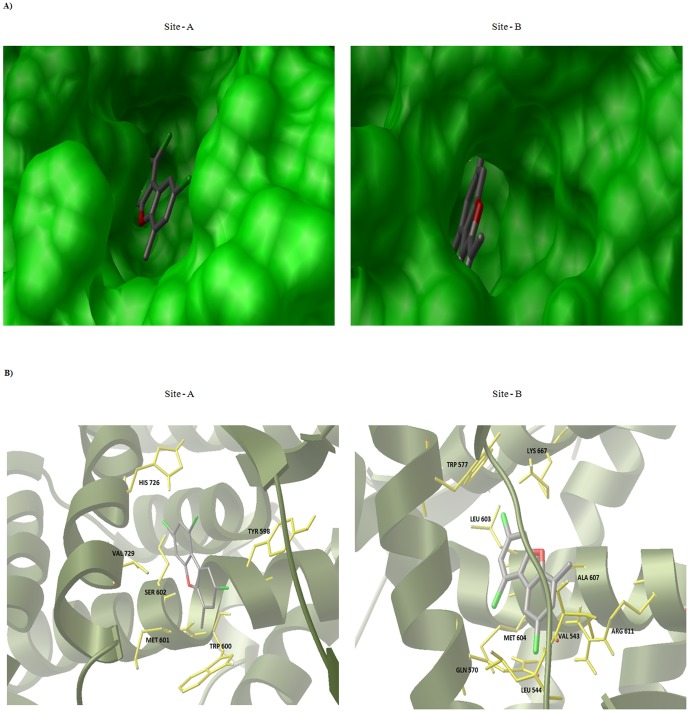
Docking of MCDF with Glucocorticoid Receptor. (A) MCDF docked in the Site –A (left side) and in the Site –B (Right side) (C) Stereo view of Site –A and Site –B important residues in Glucocorticoid Receptor. Protein is shown in cartoon, MCDF in ball-and-stick and important residues in yellow colour sticks.

By using AutoLigand and generating many different-sized fill volumes, this plot (Fig 2) revealed two curves with identifiable minima indicating potential binding sites (Site-A; plotted with red squares, Site-B; plotted with green squares) and a few sets of points (plotted in blue and pink circles) representing small cavities. Site-A has an optimal volume of 290 Å^3^ and energy per volume of −0.20182511kcal/molÅ^3^. Site-B on the other hand has an optimal volume of 536 Å^3^ and energy per volume of −0.21160837kcal/molÅ^3^. The AutoLigand fill of Site-A and Site-B for Glucocorticoid Receptor along with the bound Hexane-1, 6-Diol and the residues nearby were shown in the Fig 3a, b. In order to gain the functional and structural insight in the mechanism of interaction, molecular docking simulation of MCDF at Site-A and Site-B of Glucocorticoid Receptor was performed. Fig 4a, b illustrates the most energetically favourable binding modes of MCDF at the Site-A and Site-B of Glucocorticoid Receptor. The orientation of docking poses suggest that the residues Tyr 598, Met 601, Trp 600, Ser 602, His 726, Val 729 were occupied in the binding region of MCDF at Site-A with a free binding energy of −7.07 kcal/mol (Figure 4a) whereas the residues Val 543, Leu 544, Gln 570, Trp 577, Leu 603, Met 604, Ala 607, Arg 611, Lys 667 were occupied in the binding region of MCDF at Site-B (Figure 4b) with a free binding energy of −7.85 kcal/mol respectively. The computed inhibition constants (*Ki*) for the above binding modes are 6.55 µM for Site-A and 1.76 µM for Site-B respectively. These results suggest that MCDF can bind more effectively at Site-B compared to Site-A. Therefore, the complex of MCDF with Glucocorticoid Receptor at Site-B was selected as a representative for molecular dynamics simulations (MDS).

### Analysis of the dynamics trajectories

**Figure 5:**
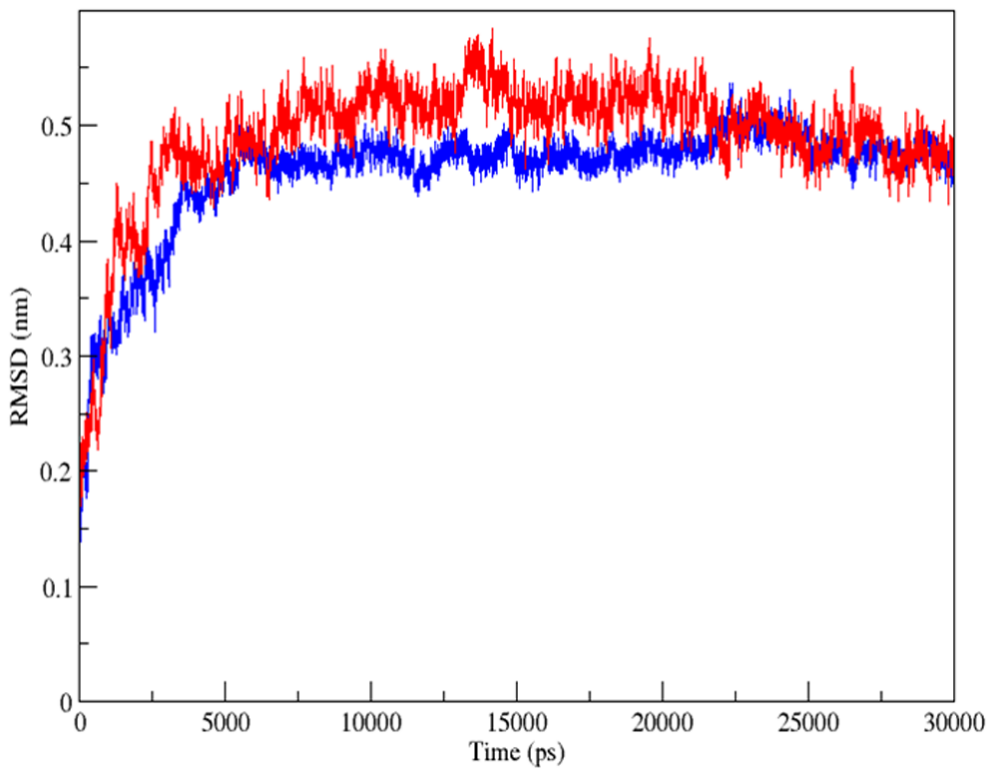
Backbone RMSD versus time plot during the 30 ns molecular dynamics simulation for Glucocorticoid Receptor (blue) and Glucocorticoid Receptor-MCDF complex (red).

In order to examine the conformational variation of Glucocorticoid Receptor (1NHZ) upon interaction with MCDF at Site-B, root-mean-square deviation (RMSD) for the backbone atoms of MCDF-1NHZ complex and 1NHZ with respect to the starting structure was calculated. Fig. 5 shows the RMSD for backbone atoms of the MCDF-1NHZ complex and 1NHZ as a function of the simulation time (30 ns). It is clearly shown that RMSD of the backbone atoms of both MCDF-1NHZ complex and 1NHZ has a same pattern of fluctuation with a steady increase in the initial 5ns followed by stability at 25ns and a steady decrease at the end of the simulation. Only a slight deviation in average backbone rmsd of MCDF-1NHZ complex was observed compared to 1NHZ indicating that the MCDF binding was stable during the MDS (Table 2).

**Table 2:** Time averaged structural properties calculated for MCDF-1NHZ complex and 1NHZ during 30 ns MDS.

	1NHZ	MCDF-1NHZ
Backbone rmsd (nm)	0.4585	0.490561
Cα-rmsf (nm)	0.132046	0.128289
Backbone Rg (nm)	1.721256	1.769971

**Figure 6:**
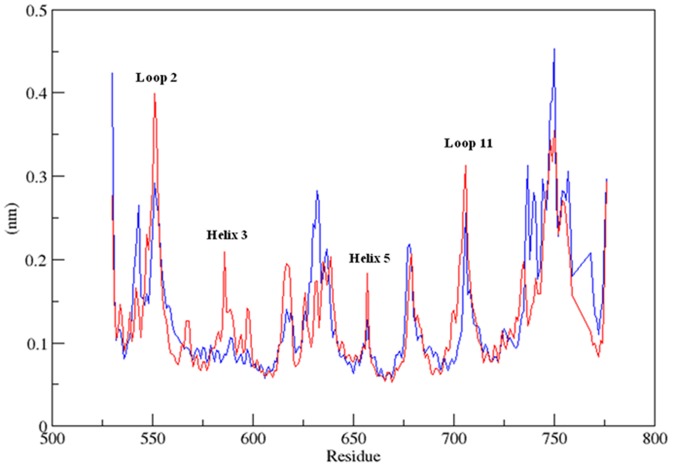
RMSF of Cα atoms as a function of amino acids for Glucocorticoid Receptor (black) and Glucocorticoid Receptor-MCDF complex (red) during 30 ns MDS.

To analyze which part of the protein was more flexible upon binding to MCDF, the root-mean-square fluctuation of the Cα atoms which is considered to be analogous to crystallographic B-factors was calculated for each residue during the MDS. Fig. 6 shows the RMSF for Cα atoms of the MCDF-1NHZ complex and 1NHZ as a function of time. Results showed that larger fluctuations were concentrated in the regions corresponding to the loop regions connecting the helices (Fig 6). Further, major fluctuations were observed in the loop region 2 (541–554), helix 3 (583–617), helix5 (638–656) and loop region 11 (706–707) indicating that binding of MCDF affected the dynamics of these regions. Labels of the respective regions of 1NHZ along with the color coding were provided in the File S2. Residues Val 543, Leu 544, Gln 570, Trp 577, Leu 603, Met 604, Aal 607 and Arg 611 that are involved in the region of MCDF-1NHZ interaction showed major fluctuations in the regions loop 2, helix 3 and helix 5. However, the average rmsf of the MCDF-1NHZ complex was less compared to the 1NHZ indicating that the MCDF-1NHZ complex was slightly stable than 1NHZ (Table 2).

**Figure 7:**
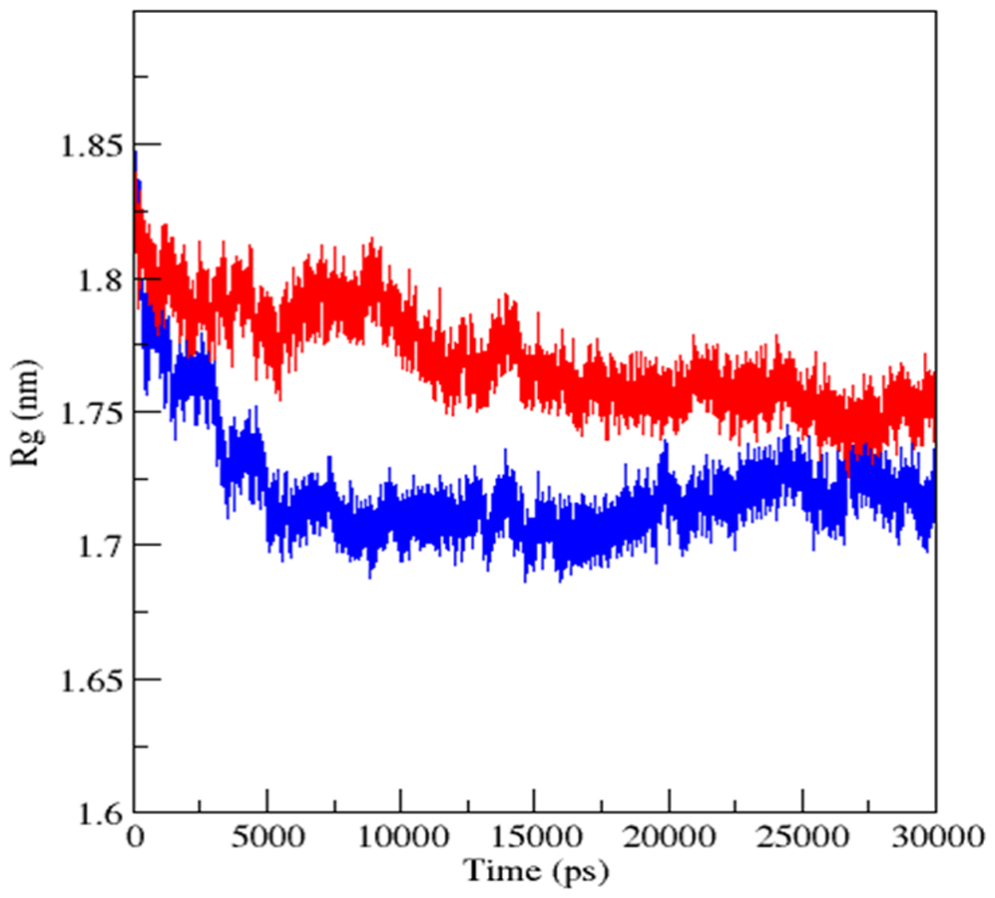
Backbone Radius of gyration (Rg) versus time plot during the 30 ns molecular dynamics simulation for Glucocorticoid Receptor (blue) and Glucocorticoid Receptor-MCDF complex (red).

**Figure 8:**
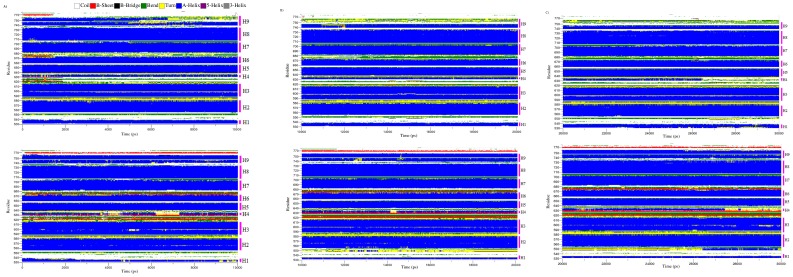
Variation of the secondary structure versus time for the Glucocorticoid receptor and Glucocorticoid Receptor.-MCDF complex during (A) 10 ns (B) 20 ns and (C) 30 ns MDS. Top represents the Glucocorticoid receptor and the bottom represents Glucocorticoid Receptor-MCDF complex.

**Figure 9:**
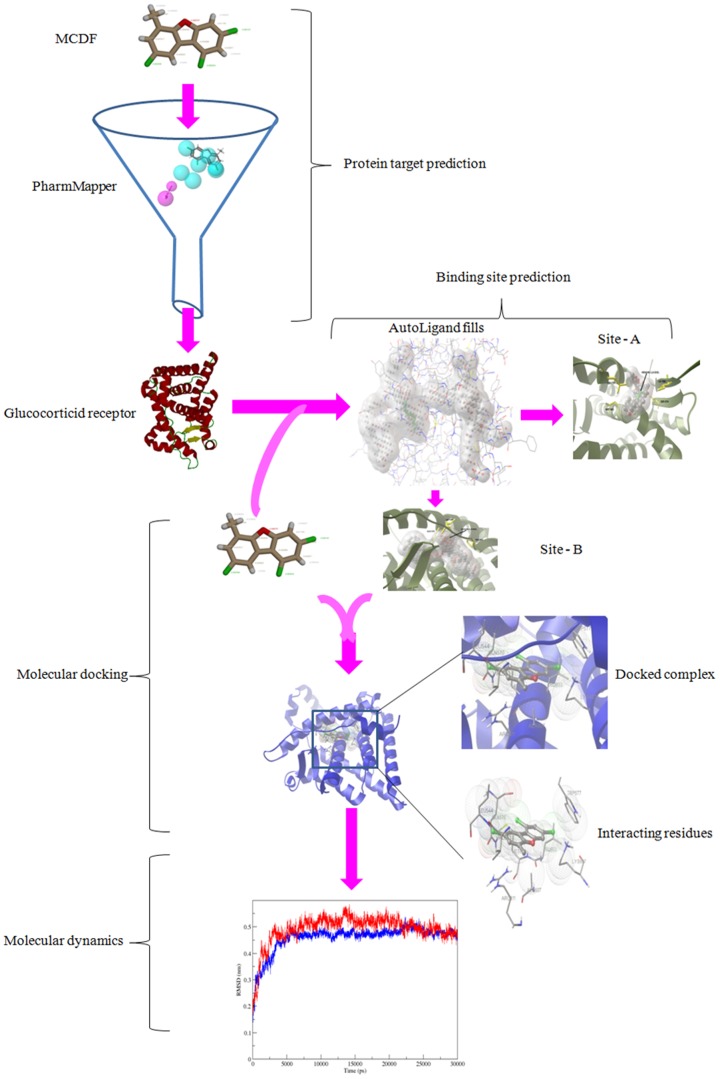
Schematic of the pipeline used in the study.

In order to investigate the local conformational stability of the system, radius of gyration (Rg) for the backbone atoms of free and bound Glucocorticoid receptor was calculated. Radius of gyration as a function of time was shown in the Fig. 7. The radius of gyration of backbone atoms showed a slight increase upon binding of the MCDF indicating a less compact structure after the simulations (Table 2, Fig. 7). Further, the analysis of the secondary structure for 1NHZ and MCDF-1NHZ complex was done using DSSP program [16]. Their respective secondary structures as a function of the time were depicted in the Fig. 8a–c. Different colours were used to distinguish between the secondary structures types. Results showed that the overall secondary structure pattern of free and bound Glucocorticoid receptor was maintained during the 30 ns MDS, though there was a slight deviations at some regions. Major changes were often seen in the helix-4 region whereas the remaining regions showed their helicity throughout the 30 ns MDS. In both free and bound Glucocorticoid receptor, residues in the helix-4 (632–636) showed fluctuating between turn and α-helix (Fig. 7a–c). Overall these DSSP analyses revealed that all the residues indeed preserve their helical content. These results showed that the docked complex structure was stable over the entire simulation time. The schematic representation of the overall study was shown in the Figure 9 given below.

## Conclusion

The day to day increase of bioactivity data has a chance to increase the success rate in the rational drug development and the present study, will offer an in depth insight into the prediction of new Breast cancer targets for MCDF using reverse pharmacophore approach. Molecular docking and molecular dynamics show that MCDF is able to interact with Glucocorticoid receptor at the active site region occupied by the residues Val 543, Leu 544, Gln 570, Trp 577, Leu 603, Met 604, Ala 607, Arg 611, Lys 667 and the interaction is stable over the simulation time. The proposed study may help to make better chemical decisions in the future.

## Supporting Information

Table S1 **Theoretical Binding protein targets for MCDF.**
(DOC)

Table S2 **Regions of INHZ. L; represents loops (green), H; represents helices (red), S; represents sheets (yellow).**
(DOC)
