# Supplementary material for: Computational Prediction and Analysis of Breast Cancer Targets for 6-Methyl-1, 3, 8-Trichlorodibenzofuran
Source: PLoS One. 2014 Nov 3;9(11):e109185. doi: 10.1371/journal.pone.0109185 (PMC4217716; doi:10.1371/journal.pone.0109185)
Supplement: Table S1 — Theoretical Binding protein targets for MCDF. (DOC) [file pone.0109185.s001.doc]

| **PDB ID** | **Molecule and Pharmacophore Model** | **Pharmacophore Color Scheme** |
| --- | --- | --- |
| 1NHZ | 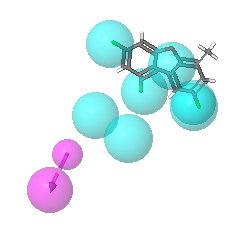 | 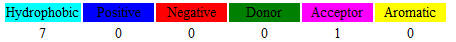 |
| 1S9J | 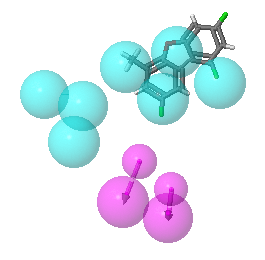 | 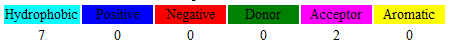 |
| 1DB1 | 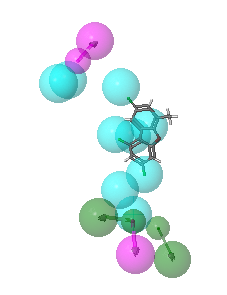 | 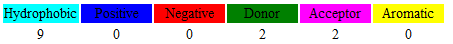 |
| 1E3K | 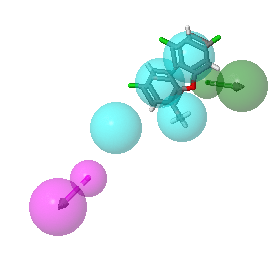 | 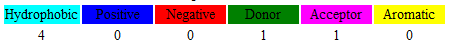 |
| 2Q2Z | 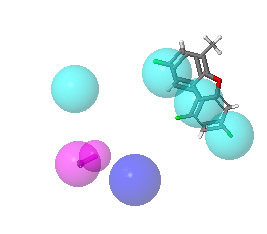 | 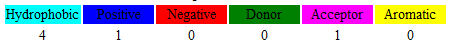 |
| 1DKF | 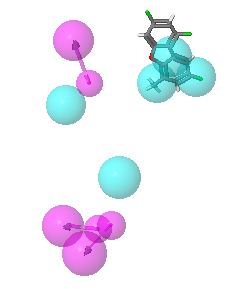 | 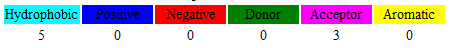 |
| 1Q3D | 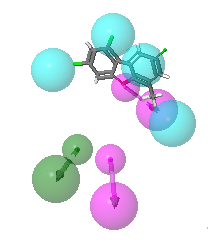 | 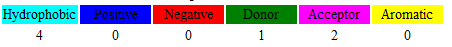 |
| 1P49 | 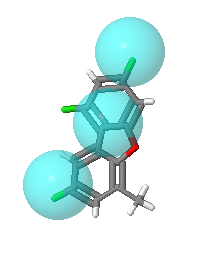 | 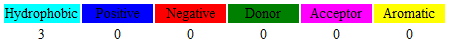 |

**Table S1: Theoretical Binding protein targets for MCDF**
