# Supplementary material for: Computational Prediction and Analysis of Breast Cancer Targets for 6-Methyl-1, 3, 8-Trichlorodibenzofuran
Source: PLoS One. 2014 Nov 3;9(11):e109185. doi: 10.1371/journal.pone.0109185 (PMC4217716; doi:10.1371/journal.pone.0109185)
Supplement: Table S2 — Regions of INHZ. L; represents loops (green), H; represents helices (red), S; represents sheets (yellow). (DOC) [file pone.0109185.s002.doc]

| **Label** | **Residue Number** | **Residues with color** |
| --- | --- | --- |
| L1 | 530 | P |
| H1 | 531-540 | TLVSLLEVIE |
| L2 | 541-554 | PEVLYAGYDSSVPD |
| H2 | 555 - 579 | STWRIMTTLNMLGGRQVIAAVKWAK |
| L3 | 580 - 582 | AIP |
| H3 | 583- 617 | GFRNLHLDDQMTLLQYSWMSLMAFALGWRSYRQSS |
| L4 | 618 - 620 | ANIL |
| S1 | 621 – 624 | LCFA |
| L5 | 625-626 | PD |
| S2 | 627-629 | LII |
| L6 | 630-631 | NE |
| H4 | 632-636 | QRMTL |
| L7 | 637 | P |
| H5 | 638-656 | DMYDQCKHMLYVSSELHRL |
| L8 | 657-658 | QV |
| H6 | 659-672 | SYEEYLCMKTLLLL |
| L9 | 673 | S |
| S3 | 674-676 | SVP |
| L10 | 677-681 | KDGLK |
| H7 | 682-705 | SQELFDEIRMTYIKELGKAIVKRE |
| L11 | 706-707 | GN |
| H8 | 708-733 | SSQNWQRFYQLTKLLDSMHEVVENLL |
| L12 | 734-738 | NYCFQ |
| H9 | 739-758 | TFLDKTMSIEFPEMLAEIIT |
| L13 | 759-768 | NN |
| S4 | 769-771 | IKK |
| L14 | 772-776 | LLFHQ |

**Table S2: Regions of INHZ.** L; represents loops (green), H; represents helices (red), S; represents sheets (yellow)
